# Riboflavin transporter deficiency: *AAV9-SLC52A2* gene therapy as a new therapeutic strategy

**DOI:** 10.3389/fncel.2025.1523773

**Published:** 2025-03-11

**Authors:** Cecilia Mei, Valentina Magliocca, Xin Chen, Keith Massey, Anai Gonzalez-Cordero, Steven J. Gray, Marco Tartaglia, Enrico Silvio Bertini, Stefania Corti, Claudia Compagnucci

**Affiliations:** ^1^Department of Pathophysiology and Transplantation (DEPT), Università degli studi di Milano, Milan, Italy; ^2^Molecular Genetics and Functional Genomics, Ospedale Pediatrico Bambino Gesù, IRCCS, Rome, Italy; ^3^Department of Pediatrics, University of Texas Southwestern Medical Center, Dallas, TX, United States; ^4^Cure RTD Foundation, Calgary, AB, Canada; ^5^Stem Cell Medicine Group, Children's Medical Research Institute, University of Sydney, Westmead, NSW, Australia; ^6^Unit of Neuromuscular and Neurodegenerative Disorders, Translational Pediatrics and Clinical Genetics, Ospedale Pediatrico Bambino Gesù, IRCCS, Rome, Italy

**Keywords:** human pluripotent stem cells, motoneuronal differentiation, neurodegenerative autosomal recessive disease, morphological neuronal phenotyping, gene therapy

## Abstract

Riboflavin transporter deficiency syndrome (RTD) is a rare childhood-onset neurodegenerative disorder caused by mutations in *SLC52A2* and *SLC52A3* genes, encoding the riboflavin (RF) transporters hRFVT2 and hRFVT3. In the present study we focused on RTD Type 2, which is due to variants in *SLC52A2* gene. There is no cure for RTD patients and, although studies have reported clinical improvements with administration of RF, an effective treatment is still unavailable. Here we tested gene augmentation therapy on RTD type 2 patient-derived motoneurons using an adeno-associated viral vector 2/9 (AAV9) carrying the human codon optimized *SLC52A2* cDNA. We optimized the *in vitro* transduction of motoneurons using sialidase treatment. Treated RTD motoneurons showed a significant increase in neurite’s length when compared to untreated samples demonstrating that AAV9-SLC52A2 gene therapy can rescue RTD motoneurons. This leads the path towards *in vivo* studies offering a potential treatment for RTD patients.

## Introduction

Riboflavin transporter deficiency (RTD), formerly known as Brown-Vialetto Van Laere syndrome, is a rare recessive neurologic condition. The disorder is a motor neuron disease characterized by defective motoneurons controlling speech, walking, swallowing, breathing and general body movements (Amir et al., 2020; O’Callaghan et al., 2019).

The syndrome is characterized by a phenotypic spectrum of *motor*, sensory, and *cranial nerve* neuropathy, resulting in muscle weakness, respiratory compromise, vision loss, sensorineural hearing loss, and sensory ataxia (Bosch et al., 2012; Foley et al., 2014; Manole et al., 2017). RTD type 2 specifically is caused by biallelic pathogenic variants in *SLC52A2* gene (Jaeger and Bosch, 2016; Johnson et al., 2012), encoding the riboflavin transporters, hRFVT2 (Foley et al., 2014; Haack et al., 2012). Riboflavin (RF) is a precursor of flavin mononucleotide (FMN) and flavin adenine dinucleotide (FAD) and reduction of its intracellular availability, through defective transporters, compromises several vital processes. RF cannot be synthesized *de novo* and is taken from the diet through riboflavin transporters hRFVT1, 2, 3, which have different tissue distribution. Specifically, hRFVT1 is preferentially expressed in the intestinal epithelium and in placenta, hRFVT2 is localized in the central and peripheral nervous system, while hRFVT3 is mainly localized in the testis, small intestine, kidney, and placenta (Jin and Yonezawa, 2022). Albeit empirical studies reported clinical improvement with the administration of RF, an effective cure is still lacking (Marioli et al., 2020; Rizzo et al., 2017).

Over the last two decades, AAV gene therapy has showed substantial improvements and benefits to patients. AAV vectors have emerged as one of the safest and most used vectors for gene replacement (Kantor et al., 2014). Specific targeting capabilities conferring a variety of capsid choice have made recombinant AAV the ideal vector used for gene delivery to many tissues and organs, including the central nervous system (CNS) (Hocquemiller et al., 2016; Ling et al., 2023). Furthermore, AAV exhibit a stable transgene expression in post-mitotic cells, neuronal tropism, low immunogenicity (Murlidharan et al., 2014). The serotype 9 (AAV9) is able to cross the blood–brain barrier with high transduction efficacy, representing a good vector for intravascular administration (Saraiva et al., 2016). The FDA approved Onasemnogene abeparvovec (Zolgensma) as a AAV9 gene therapy for infants with spinal muscular atrophy (SMA) (Ogbonmide et al., 2023).

Recombinant AAV9 vectors display widespread transduction in animals, from mice to larger animal models (Choudhury et al., 2017; Karumuthil-Melethil et al., 2016; Federici et al., 2012; Gray et al., 2013; Snyder et al., 2011; Masamizu et al., 2011; Bucher et al., 2013; Haurigot et al., 2013; Samaranch et al., 2013), but often present low transduction efficiency in cells *in vitro* (Gao et al., 2004; Zincarelli et al., 2008).

We previously generated and characterized several induced pluripotent stem cells (iPSCs) lines generated from RTD patients with different variants in the *SLC52A2* gene. Specifically, we assessed their molecular (i.e., antioxidant response), morphological (i.e., neurite’s length) and functional (i.e., calcium metabolism) features (Niceforo et al., 2021; Colasuonno et al., 2020). Data collected in previous studies suggest that RF supplementation partially rescues the RTD phenotype, and the combined treatment of RF plus antioxidants provides further improvements of these biomarkers.

In this study we confirmed the morphological phenotype of RTD iPSC-derived motoneurons showing a shorter neurite’s length. In parallel we demonstrated that sialidase treatment increased *in vitro* transduction of AAVs. Finally, we demonstrated the potential of gene therapy to rescue RTD neurodegeneration. Since all aspects of RTD type 2 disease stem from the loss of *SLC52A2* gene function, gene replacement therapy represents a reasonable and promising approach to provide a meaningful benefit for RTD patients.

## Materials and methods

### Clinical information

Clinical features of RTD P1 (carrying the variants c.155C > T (p.Ser52Phe) and c.935 T > C (p.Leu312Pro)) had been previously reported (Niceforo et al., 2021). In particular, thanks to the timely treatment with riboflavin (75 mg/kg QID) and antioxidant therapy at 2.5 years of age she has remained neurologically stable.

RTD P2 (carrying the variants c.505C > T (p.Arg169Cys) and c.1030_1031del (p.Leu344Alafs*100)) is a girl presenting the first symptoms (arm weakness and dysphagia) at 8 months of age. At 12 months old she was diagnosed reporting severe optic atrophy, bilateral sensorineural deafness, sensory neuropathy and complete diaphragm paralysis (Magliocca et al., 2024).

### Patient-derived iPSCs

The studies were conducted in compliance with the Code of Ethics of the World Medical Association (Declaration of Helsinki), and with national legislation and institutional guidelines (local institutional ethical committee, Ref. 1410_OPBG_2021, date of approval 11 February 2019). Informed consent was obtained from the subjects involved in the study. Patient skin fibroblasts were cultured in Dulbecco’s Modified Eagle Medium (Sigma Aldrich, Cod D5671), supplemented with 10% of Fetal Bovine Serum (Gibco Cod 10082-147) and penicillin/streptomycin (Gibco, Cod 15140148) at 37°C, 5% CO_2_ and 21% O_2_. When the cells reached 80% of confluence, they were reprogrammed as described (Okita et al., 2011).

**Figure 1:**
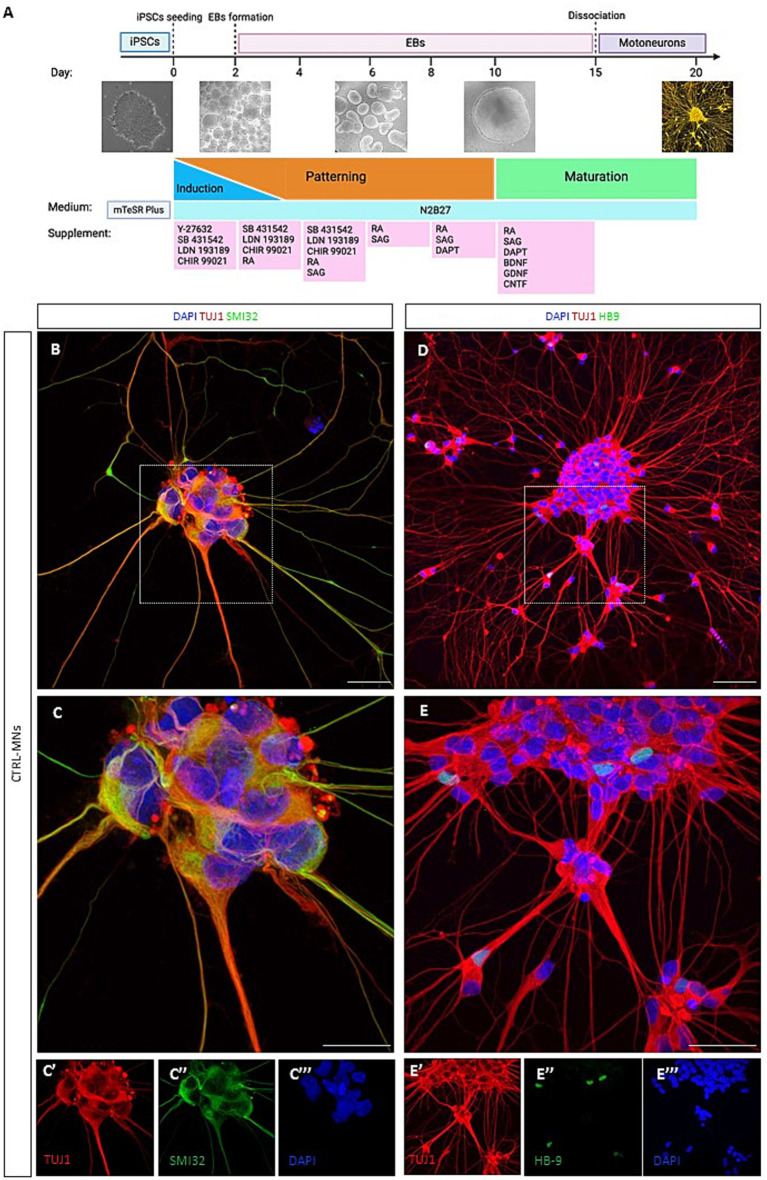
3D/2D neural differentiation and characterization of iPSC-derived motoneurons. **(A)** Schematic showing a stepwise differentiation of motoneurons using the optimized 3D/2D protocol, showing EBs formation and the mature 2D motoneurons. Created with Biorender.com. **(B)** Day 20 immunofluorescence confocal image analysis for the neural marker TUJ1 (in red) and SMI-32 (in green). **(C)** High-magnification confocal image of control motoneurons co-stained for TUJ1 and SMI32 with images showing separate color channels (C′, C″, C″’). **(D)** Representative image of neurons positive for TUJ1 (in red) and HB9 (in green). **(E)** High-magnification confocal image of TUJ1 and HB9positive control motoneurons with images showing separate color channels (E’, E,” E”’). Nuclei counterstained with DAPI. Scale bar = 50um.

**Figure 2:**
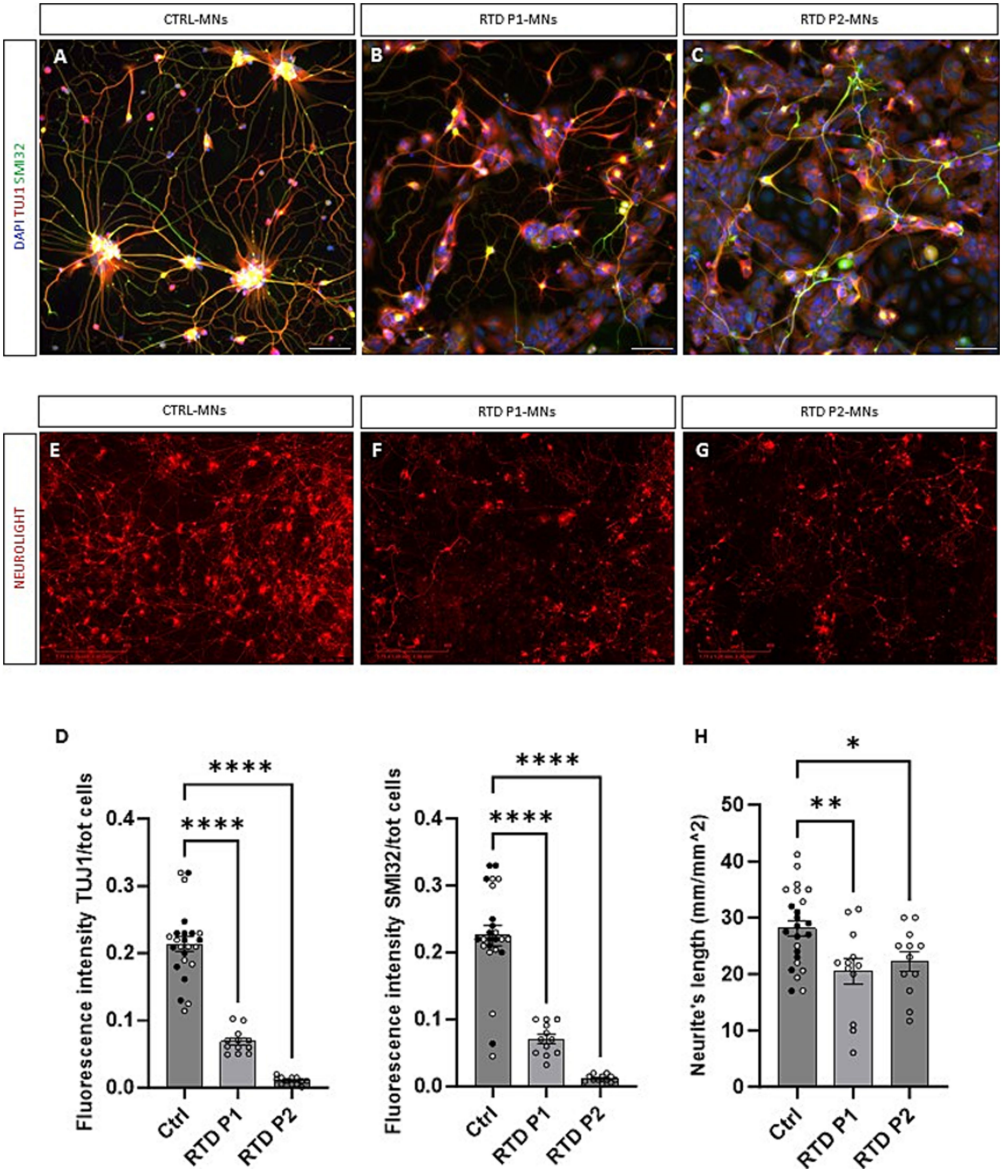
Morphological characterization of RTD motoneurons. **(A–C)** Representative images of control and RTD motoneurons positive for TUJ1 (in red) and SMI32 (in green). **(D)** Quantification of TUJ1 and SMI32 fluorescent signals demonstrated a significant decrease in fluorescence intensity in RTD iPSC-derived motoneurons (****p < 0.0001, one-way ANOVA; mean ± SEM; *N* = 3 independent experiments). **(E–G)** Neurites’ length measurement of RTD and control motoneurons positive for Neurolight red lentivirus in the Incucyte system at day 28. **(H)** Quantification confirmed a significant reduction of neurite extension in both lines of RTD motoneurons (*p < 0.05, **p < 0.01, one-way ANOVA; mean ± SEM; *N* = 3 independent experiments). Nuclei co-stained with DAPI in **(A–C)**. The CTRL bar includes data points from two CTRL cell lines (one represented by black dots, the other by white dots). Scale bar = 50 um in **(A–C)**. Scale bar = 400 um in **(E–G)**.

Pluripotency characterization of RTD P1 and RTD P2 iPSC lines had been previously reported (Rizzo et al., 2017; Okita et al., 2011). Clinical features and characterization of RTD P1 and P2 iPSC lines had previously been reported (Rizzo et al., 2017; Okita et al., 2011). Control iPSCs (CTRL iPSCs) were obtained from healthy individual from System Biosciences Coriell (GM23338 and AG28869) and were derived from fibroblasts of a healthy individual using non-integrating episomal technology. Both control lines were used for experiments shown in Figures 1, 2, while for the transduction experiments only one control line (GM23338) was used.

### Maintenance and differentiation of induced pluripotent stem cells (iPSCs) into embryoid bodies (EBs) to develop motoneurons

RTD iPSCs and CTRL iPSCs (DIV 6–8) were detached with Accutase (Sigma-Aldrich, Cod SCR005) for 5 min at 37°C. Then, single cells were cultured on ultra-low attachment dishes, using the Basal Medium N2/B27 consisting of: DMEM/F12 (Sigma Aldrich, Cod D0697), Neurobasal (Gibco, Cod 21103049), Glutamax (Gibco, Cod 35050061), Pen-strep (Gibco, Cod 15140122), B27 supplement minus Vit A, (Life technologies, Cod 12587010), N2 supplement (Life technologies, Cod 17502-048) and 2-ME (Gibco, Cod 21985-023). At Day 0, the medium was supplemented with Y27632 (10 uM) (Cell Signaling, Cod 13624) LDN193189 (0.1 uM) (StemCell, Cod 72149), SB431542 (20uM) (StemCell, Cod S4317) and CHIR 99021 (3 uM) (Sigma-Aldrich, Cod SML1046). At Day 2, the EBs were resuspended in the previous medium plus RA (100 nM) (Sigma Aldrich, Cod R2625) and no Y27632 was added. Then, at day 4, SAG (500 nM) (Sigma-Aldrich, Cod 566660) was added; at day 7, the EBs were resuspended in N2B27 plus RA and SAG. At day 9, DAPT (10 uM) (StemCell, Cod 72082) was added and at day 11 the EBs were resuspended in N2B27 plus RA, SAG, DAPT, BDNF (10 ng/mL), GDNF (10 ng/mL), and CNTF (10 ng/mL) (Sigma-Aldrich, Cod C3710). At day 15, the EBs were collected in a tube, washed in PBS and rinsed with Trypsin–EDTA 1X (Euroclone, Cod ECB3052) plus DNAse (20 ug/mL) (Thermo Fisher Scientific, Cod AM2235). Tubes were then placed in warm bath for 15 min and then EBs were dissociated to become a single cell suspension and FBS was added to neutralize the trypsin. Cells were then centrifuged for 5 min at 300 g and, after the removal of the supernatant, they were passed through a 70-um strainer. After the dissociation process, MNs were plated for further analyses.

### AAV vectors

The AAV9-GFP vector design has been previously described (Gray et al., 2011). The AAV9-SLC52A2 vector was developed similarly, but with a moderate strong UsP promoter (Chen et al., 2023) and bovine growth hormone (BGH) polyadenylation signal to drive expression of a codon-optimized human *SLC52A2* sequence. Codon optimization was carried out by ATUM (Menlo Park, CA, USA). Both AAV vectors used a self-complementary genome configuration. The AAV vectors were manufactured by the University of North Carolina Vector core, according to published methods (Grieger et al., 2016).

### Pre-treatment with Sialidase and AAV9 transduction

**Figure 3:**
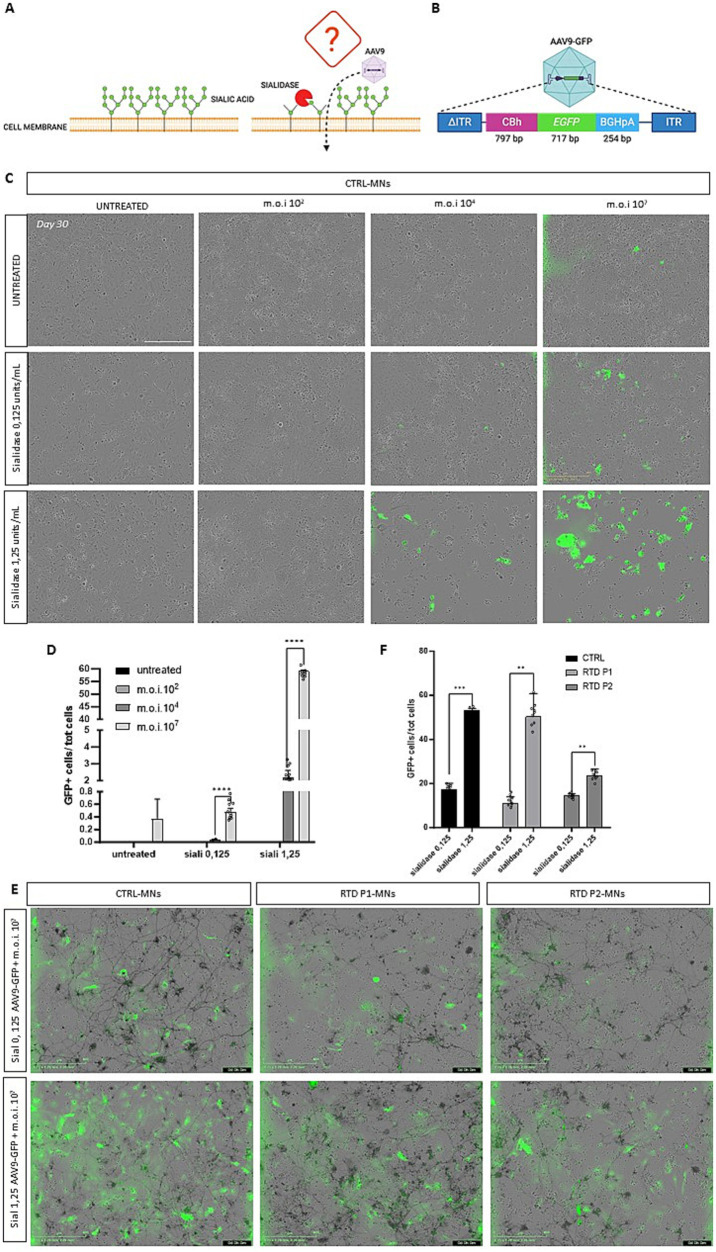
Sialidase treatment and the effect on AAV9-GFP vector transduction. **(A)** Schematic of sialidase mechanism. Removal of siliac acids from the cells surface, might increase the infectivity of the virus. **(B)** Schematic drawing of AAV9-CBh-GFP viral vector construct. **(A,B)** Created with Biorender.com. **(C)** Representative images of day 30 motoneurons after sialidase treatment and after transduction with 10 (O’Callaghan et al., 2019); 10^4^ and 10^7^ vg/cell MOI GFP expression shows cells that have been transduced. **(D)** Graph showing the percentage of GFP transduced motoneurons (****p < 0.0318, Kruskal-Wallis; mean ± SEM; *N* = 3 independent experiments). **(E)** Representative images of motoneurons pre-treated with sialidase and then transduced with the AAV9-GFP vector. **(F)** Graphs showing the percentage of GFP-positive cells demonstrate a significant increase in transduced cells with 1.25 U/mL sialidase treatment (*** *p* < 0.0001, **p < 0.001, unpaired t test; mean ± SEM, *N* = 3 independent experiments). Scale bar 400 um in **(C,E)**.

To increase the infectivity of the virus, Sialidase (Roche, cod. 10269611001) was used. After the EBs dissociation, motoneurons were left to adhere on a precoated plates with poly-O-ornithine (50 ug/mL) and laminin (20 ug/mL) for two days. Then, cells were washed 3 times with PBS and pretreated with Sialidase at 2 different concentrations: 0.125 U/mL and 1.25 U/mL for 3 h at 37°C. Following washing with PBS (three times), cells were transduced with AAV9-GFP or AAV9-SLC52A2 in a concentration of 10^7^ m.o.i for 24 h. The following day, the cell media was completely removed and replaced with the fresh media. Then, cell media was changed every other day until day 40 (Figure 3).

### Immunofluorescence assay

Mature RTD and control MNs were plated on coverslips placed in a pre-coated 24-well plate with poly-O-ornithine (50 ug/mL) and laminin (20 ug/mL). After 5 days, MNs were fixed with 4% paraformaldehyde for 10 min at RT. BSA 5% blocking solution was used and a 0.1% Triton X-100 was added (Sigma) for permeabilization. Incubation was performed with the primary antibody against TUJ1 (Sigma Aldrich, Cod 8578) diluted 1:500 and maintained at RT for 2 h, anti-SMI32 (Sigma Aldrich, Cod N4142) diluted 1:200 O/N at 4°C and anti HB-9 (Invitrogen, Cod PA5-23407). AlexaFluor 555 (Thermo Fisher Scientific, Cod A21425) and AlexaFluor 488 (Thermo Fisher Scientific, Cod A11070) were used as secondary antibodies, diluted 1:500 and incubated at RT for 1 h. Nuclei were counterstained using DAPI (Thermo Fisher Scientific, Cod. D1306). Finally, the cells were observed with a confocal Leica Dmi8 fluorescence microscope (Leica Microsystems, Germany) and acquired images were digitally elaborated with a modular image processing and analysis software (LasX Software, Leica).

### Neurites’ length assay with the Incucyte system

**Figure 4:**
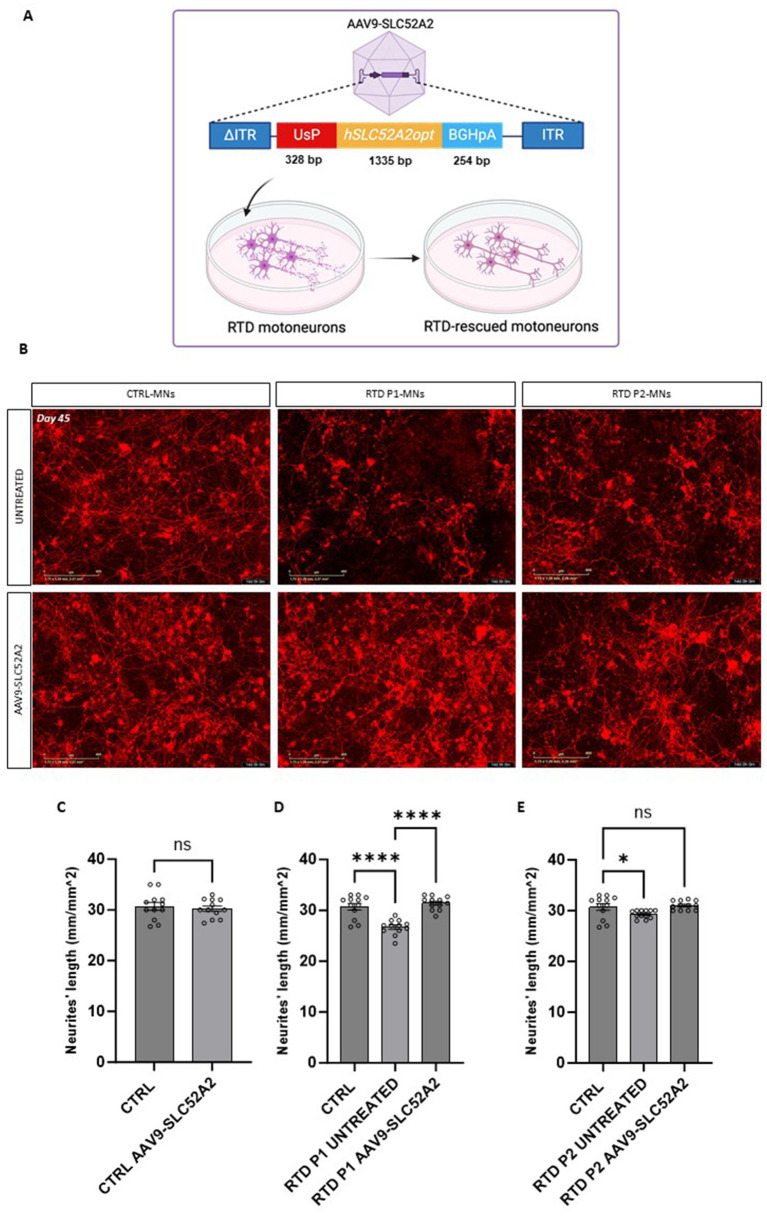
SLC52A2 gene therapy rescues RTD motoneurons neurites morphology. **(A)** Schematic of gene therapy construct. Created with Biorender.com. **(B)** Representative Incucyte image of the neural network formation of CTRL and RTD motoneurons (positive for Neurolight lentivirus in red) following gene therapy. **(C–E)** Graphs depicting the quantification of neurites’ length at day 45 of differentiation in treated and untreated motoneurons (****p < 0.0001, *p < 0.01, one-way ANOVA; mean ± SEM; *N* = 3 independent experiments). Scale bar 400 um.

**Figure 5:**
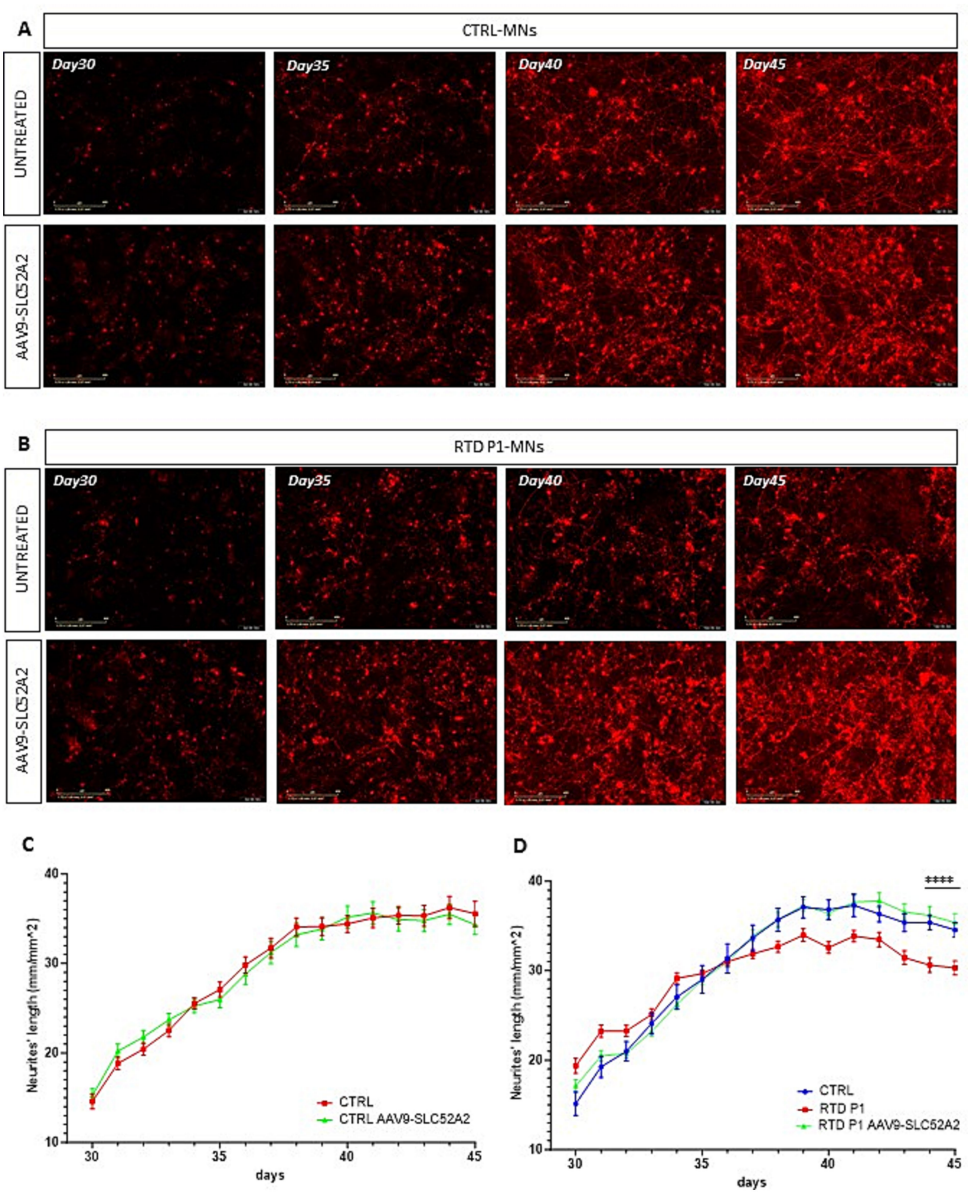
Time-laps imaging of the neurites’ length in untreated and treated motoneurons with AAV9-SLC52A2 vector. **(A,B)** Representative fluorescent images of Neurolight red positive neurons of CTRL and RTD P1 at different days of neuronal differentiation, showing that at the end of *in vitro* neurogenesis (day 45), motoneurons of RTD patient showed longer neurites compared to those untreated. **(C,D)** Graphs showing neurites’ length of CTRL and RTD P1 over *in vitro* neurogenesis (**p = 0.001, one-way ANOVA; mean ± SEM, *N* = 3 independent experiments, *N* = 4 images per independent experiment per sample). Scale bar 400 um.

Motoneurons were plated at a density of 5′000 cells/well in a Matrigel precoated 96-well plate (TPP, Cod 92696) and neurites’ length was measured using the IncuCyte System (Sartorius, Essen BioScience) with the Neurite Analysis application for Neurolight labeled cells. Cells were transduced with a lentiviral-based vector encoding the Incucyte Neurolight Lentivirus (Sartorius, Essen BioScience, Cod 4807) following manufacturer’s instructions (Figure 4). Live imaging experiments (in Figure 5) were performed by acquiring every 4 h for 15 days, from the 25th to the 40th day of the neural differentiation process. Phase-contrast and fluorescent images were acquired for every experiment. Analysis parameters for NeuroTrack software module-processing definitions were optimized individually for each experiment according to the workflow outlined in the manufacturer’s manual. Microplate graphs were generated using the time plot feature in the graph/export menu of the Incucyte SX5 software. Raw data of neurites’ lengths were exported to Microsoft Excel and GraphPad Prism to calculate mean values ± SEM.

### Statistical analysis

Raw data were collected from each individual experiment of three independent biological replicates. The data were analyzed as the mean ± the standard deviation (SD) or the standard error of the mean (SEM). Significance was tested using ordinary unpaired t test (when two sample groups were compared) or one-way ANOVA (parametric test) (when more than two sample groups were compared) for normally distributed data. For data that do not passed the normality test, Kruskal Wallis (non parametric) test has been used to calculate statistical significance. GraphPad-Prism software (v9.3.1, GraphPad Software) was used to statistically analyze the data.

## Results

### Characterization of motoneurons derived from 3D/2D neural differentiation protocol

We first tested two protocols for the generation of mature motoneurons using a control iPSC line (Corti et al., 2012). Using a 2D adherent differentiation protocol neurons expressed the pan-neural marker TUJ1 but failed to show the mature motoneuron marker HB9 (Supplementary Figures S1C,D) even at day 30 in culture. iPSCs were successfully differentiated into motoneurons using an adapted 3D/2D protocol (Figure 1A; Maury et al., 2014). This directed differentiation protocol is based on the generation of embryoid bodies (EBs), which at later stages are dissociated to obtain pure cultures of motoneurons (90 ± 1.49%) (Supplementary Figure S1E).

At day 20 *in vitro* neural differentiation was confirmed by immunopositivity of the pan-neural marker TUJ1 and SMI32 (Figures 1B,C). We further assessed for motoneuron maturation using the post-mitotic motoneuronal marker HB9 (Figures 1D,E). Results confirmed the ability of the 3D/2D protocol to generate mature motoneurons being positive for the markers TUJ1, SMI32 and HB9.

### Characterization of RTD-derived motoneurons

Next, we differentiated two iPSC lines from healthy donors (CTRL) and two iPSC lines from RTD patients, carrying different mutations (RTD P1 *155C > T;935 T > C* and RTD P2 *1030_31 del;505C > T*) in the *SLC52A2* gene. RTD motoneurons expressed TUJ1 and SMI32 proteins, however formation of neuronal networks was less evident (Figures 2A–C). A significant decrease in fluorescence intensity was observed in RTD iPSC-derived motoneurons (Figure 2D, **** *p* < 0.0001, one-way ANOVA; mean ± SEM; *N* = 3 independent experiments, *N* = 3 images per independent experiment per sample), confirming the impaired neuronal differentiation demonstrated using a different methodology (see Conclusions). Next, we sought to confirm if neuronal networks were impaired in diseased motoneurons. Day 20 motoneurons were cultured on 96-well plates for live imaging and transduced using a Neurolight Red Lentivirus (Incucyte SX5 system) which enabled highly efficient and non-disruptive labeling of iPSC-derived neurons (Figures 2E–G). Fourteen days following transduction RTD motoneurons showed a significant reduction in neurite extension (Figure 2H, ****p = <0.0001, one-way ANOVA; mean ± SEM; *N* = 3 independent experiments, *N* = 4 for each independent experiment per sample). These data demonstrate that using 3D/2D neural differentiation RTD motoneurons exhibit disrupted neurite extension starting to elucidate characteristics of disease *in vitro.*

### Sialidase treatment increases transduction efficiency of AAV9 in iPSC-derived motoneurons

Efficient gene transfer by AAV9 vectors requires an atypical interaction with non-sialylated cell surface glycans (Shen et al., 2011; Bell et al., 2011). We sought to develop an effective AAV gene therapy for RTD by testing whether sialidase treatment, which removes the siliac acid from the cell surface increases AAV9 transduction (Figure 3A). Different multiplicity of infection (MOI) of a control AAV9-CBh-GFP vector (Figure 3B) was used to assess transduction efficiency in motoneurons following sialidase treatment at various concentrations (Figure 3C). Transduction efficiency was analyzed 5 days post-transduction at day 30 in culture. MOI 10^2^ was too low to detect any GFP positive cells even in both untreated and sialidase treated cells. However, treatment at both 0.125 and 1.25 units/mL significantly increase transduction when compared to untreated control at moi 10^4^ (****p < 0.0001, Kruskal-Wallis; mean ± SEM; *N* = 3 independent experiments, *N* = 4 images per independent experiment per sample). AAV9 vector at 10^7^ moi significantly outperformed all other treatments (Figure 3D, ****p < 0.0318, Kruskal-Wallis; mean ± SEM; *n* = 3 images, *N* = 3 independent experiments). Finally, to evaluate if transduction varies between control and RTD motoneurons we tested the transduction efficiency of the AAV9-CBh-GFP vector in control and RTD motoneurons pre-treated with 0.125 and 1.25 u/mL sialidase. A significant increase in the percentage of GFP motoneurons in control, RTD P1 and P2 was observed in the 1.25 u/mL sialidase treated motoneurons (Figures 3E,F, *** *p* < 0.0001, ** *p* = 0.0033 for RTD P1, **p = 0.0078 for RTD P2, unpaired t-test; mean ± SEM; *N* = 3 independent experiments, *N* = 3 images per independent experiment per sample).

### Gene therapy successfully rescues RTD motoneurons neurites length

Next, to treat RTD motoneurons, we designed a AAV9-UsP-SCL52A2 vector carrying the human codon optimized *SLC52A2* cDNA (Figure 4A).

We transduced RTD P1 and P2 motoneurons, with the AAV9-UsP-SLC52A2 at day 25 in culture using the optimized 1.25 u/mL sialidase treatment and m.o.i of 10^7^ and evaluated motoneurons at day 45 in culture. To establish the effects of the gene therapy on the neurites’ length disease phenotype we also transduced motoneurons with the Neurolight red lentivirus (Figure 4B; Supplementary Video S1) for the automated measurement of the neurite’s length (Supplementary Figure S2). Importantly, following AAV treatment, we found that neurites’ length of RTD P1 and P2 motoneurons were significantly longer than untreated (Figures 4C–E, *p* = 0.5011 for CTRL, **p = 0.0050 for RTD P1, *p = 0.0192 for RTD P2, ANOVA; mean ± SEM; *N* = 3 independent experiments, *N* = 4 images per independent experiment per sample). Furthermore, length was restored to levels similar to control MNs (Figures 4D,E). A more pronounced rescue was observed in treated RTD P1 motoneurons, therefore to evaluate the effect of the gene therapy on neurogenesis, we imaged untreated and treated control and RTD P1 motoneurons at several time points (days 30, 35, 40 and 45 of neuronal differentiation) (Figures 5A,B). We showed that in untreated RTD motoneurons, a presumptive neural network was able to form, but over time, motoneurons neurites were lost and cells underwent neurodegeneration (Figures 5A,B; Supplementary Video S1). Meanwhile, following gene therapy with the AAV9-SLC52A2 vector the neural network in RTD patient was preserved over time, showing a progressive amelioration of the neurites’ length in transduced RTD motoneurons (Figure 5C, **p = 0.001, ANOVA; mean ± SEM; *N* = 3 independent experiments, *N* = 4 images per independent experiment per sample). No changes in terms of neurites’ length nor network formation/degeneration were observed in control motoneurons treated with AAV9- UsP-SLC52A2 indicating the overexpression of *SLC52A2* is not detrimental to neurons *in vitro* (Figure 5C).

## Conclusion

*In vitro* stem cell-derived motoneurons are a valuable tool to analyse the pathomechanisms underlying motoneuron diseases. In this study, we demonstrated the efficient differentiation of iPSCs into motoneurons by adapting a previously described protocol (Maury et al., 2014). Various types of neuronal cultures have been described to date, and although this model has been broadly used, the majority of the models generate immature motoneurons (Bucchia et al., 2018). Here we showed that our iPSC-derived motoneurons matured and expressed the post-mitotic marker HB9 enabling proper RTD modelling *in vitro.* Despite the improved methodology used in this study to obtain iPSC-derived motoneurons, the fluorescent intensities of TUJ1 and SMI32 are significantly decreased in RTD motoneurons (Figure 2D), thus confirming the impaired neuronal differentiation of RTD cells as previously demonstrated using different neuronal differentiation methodology (Marioli et al., 2020; Rizzo et al., 2017; Niceforo et al., 2021). Additionally, we do not exclude that the decreased TUJ1 and SMI32 levels in RTD motoneurons are a consequence of impaired neuronal survival of RTD motoneurons, since neuronal apoptosis has been recently demonstrated as a contributing event in RTD pathogenesis (Marioli et al., 2024).

One of the most compromised features in RTD motoneurons is the cytoskeleton (Marioli et al., 2020; Rizzo et al., 2017; Niceforo et al., 2021). Our morphometric analyses of RTD iPSC-derived motoneurons confirmed that RTD disease leads to neurites that are significantly shorter than those of the healthy motoneurons (Marioli et al., 2020; Rizzo et al., 2017; Niceforo et al., 2021).

Previous studies have reported that gene therapy testing in iPSC-derived motoneurons is limited due to low transduction efficiency by AAV vectors. Here, we described that treatment with Sialidase significantly increased cell surface binding and infectivity of adeno-associated virus (AAV) serotype 9 (Shen et al., 2011). We also established the optimal experimental condition to obtain the maximal rate of infection. Various combinations of sialidase and MOI vector allowed to obtain more than the 50% of transduction efficiency.

The majority of RTD patients are responsive to RF supplementation, however, it remains unclear if this supplementation will be effective in preventing symptoms for life in responsive patients, or simply delay the occurrence of symptoms (Jaeger and Bosch, 2016; Koy et al., 2012). It is therefore necessary to look for a strategy that can alleviate the sufferings of all RTD patients. Considering the recent advances in the field of gene therapy, we designed and tested a AAV9-SLC52A2 gene supplementation therapy which would be efficacious on all RTD patients independently to the carried mutations and/or their responsiveness to RF treatment.

In the present study, we aimed at promoting the expression of the correct SLC52A2 protein by introducing it by AAV9-SLC52A2 transduction. Promisingly, gene therapy was able to restore neurites length of RTD motoneurons generating long neurites maintaining a robust neural network during the neurogenesis compared to the untreated RTD motoneurons, which resulted in a breakable and fragile neuronal network undergoing degradation over time.

Collectively, our results indicate that *AAV9-SLC52A2* vector rescues the neural phenotype in motoneurons derived from RTD Type 2 patient iPSCs, which warrants further *in vitro* as well as *in vivo* studies to develop gene therapy as a potential clinical treatment for these patients. In fact, we observed recovery of the morphological abnormalities of the RTD neurites and we are currently working on the evaluation of the functional aspects of AAV9-SLC52A2 transduced RTD neuronal cells. Recently, several *in vivo* viral-vector gene therapies are currently on the market and the field of gene therapy for neurological conditions is continuously evolving (Ling et al., 2023). For many years, rare neurodegenerative disorders as amyotrophic lateral sclerosis (SLA), spinal muscular atrophy (SMA) and RTD were considered incurable, but now, gene therapy might offer an effective treatment. Currently, many research efforts are focused on finding new therapeutic strategies that would treat RTD independently from patients’ variants. These findings, offer the first insights into gene therapy efficacy for RTD patients worldwide.

## Data Availability

The data presented in this study have been deposited in Zenodo with the following doi: 10.5281/zenodo.14916787.
