# Supplementary material for: Riboflavin transporter deficiency: AAV9-SLC52A2 gene therapy as a new therapeutic strategy
Source: Front Cell Neurosci. 2025 Mar 11;19:1523773. doi: 10.3389/fncel.2025.1523773 (PMC11933037; doi:10.3389/fncel.2025.1523773)
Supplement: Supplementary file 1 [file Data_Sheet_1.pdf]

Figure S1.

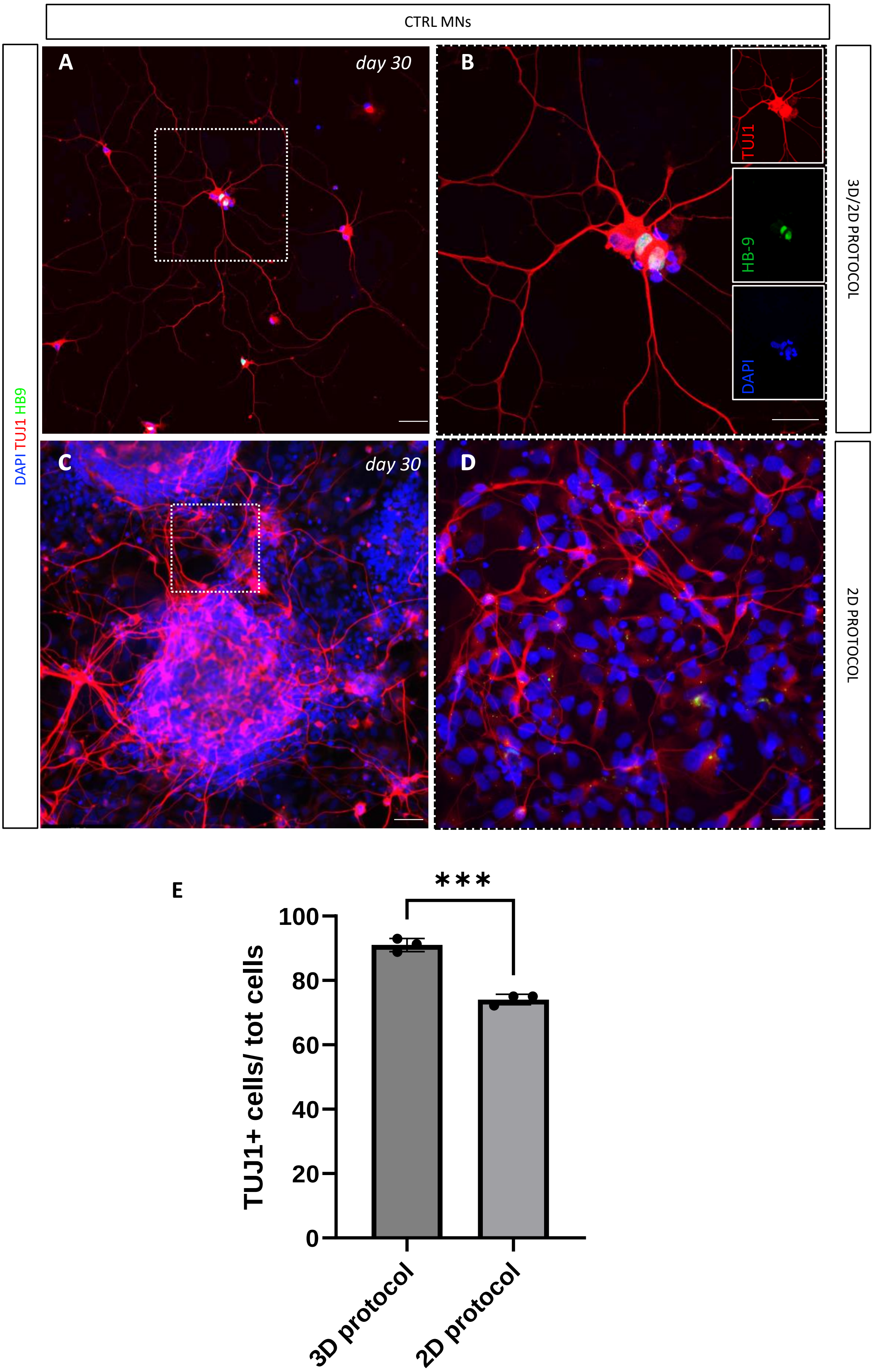

Figure S1. **Neural differentiation efficiency comparing 2D and 3D/2D protocol.** (A) Representative images of control motoneurons positive for the neural marker TUJ1 and HB9 following the 3D/2D protocol. (B) High-magnification confocal image of TUJ1+/HB9+ control motoneurons with single channel images on the right). (C) The 2D protocol was able to generate only TUJ1+ control motoneurons; no HB9+ cells were observed. (D) High-magnification of TUJ1+/HB9- control motoneurons. (E) Quantification shows the percentage of TUJ1+ cells on the total of the cells (DAPI count) confirming an efficiency of 90% ± 1.49 of the 3D/2D protocol compared with the 73% ± 1.49 efficiency of 2D protocol (*** $p < 0.0001$ unpaired t test; mean ± SEM; n= 3 images, N=3 independent experiments, N= 3 images per independent experiment per sample). Nuclei co-stained with DAPI. Scale bar 50um.

Figure S2.

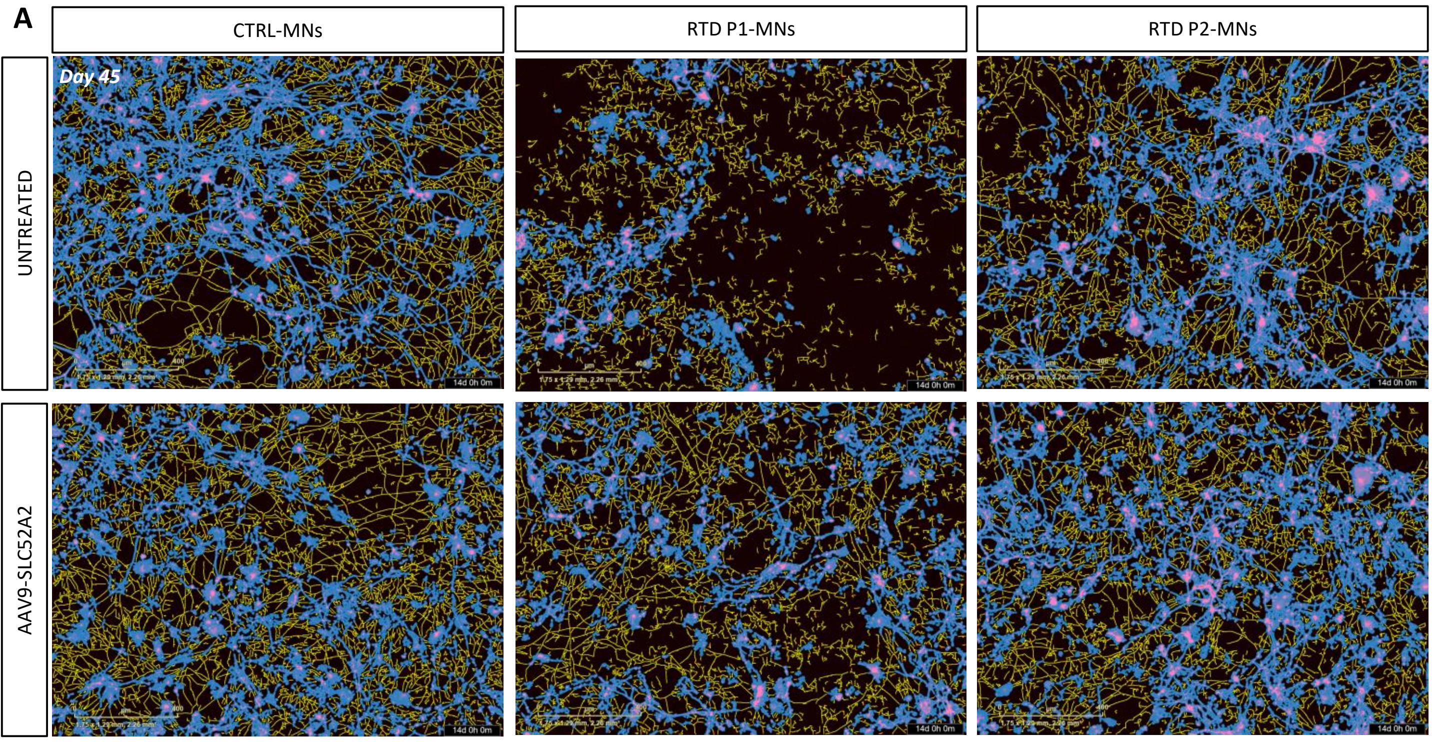

Figure S2. Depiction of the neural mask matching 45 day motoneurons neurites from **figure 4B** using Color NeuroTrack software in the Incucyte SX5 system. Neurites are depicted in yellow, and the cellular body clusters in blue. Scale bar 400 um.
